# *MC4R* Gene Polymorphisms Interact With the Urbanized Living Environment on Obesity: Results From the Yi Migrant Study

**DOI:** 10.3389/fgene.2022.849138

**Published:** 2022-04-14

**Authors:** Ye Wang, Li Pan, Shaoping Wan, Wuli Yihuo, Fang Yang, Huijing He, Zheng Li, Zhengping Yong, Guangliang Shan

**Affiliations:** ^1^ School of Population Medicine and Public Health, Chinese Academy of Medical Sciences and Peking Union Medical College, Beijing, China; ^2^ Department of Epidemiology and Statistics, Institute of Basic Medical Sciences, Chinese Academy of Medical Sciences, School of Basic Medicine, Peking Union Medical College, Beijing, China; ^3^ Sichuan Cancer Hospital and Institute, Sichuan Cancer Center, School of Medicine, University of Electronic Science and Technology of China, Chengdu, China; ^4^ Puge Center for Disease Control and Prevention, Liangshan, China; ^5^ Xichang Center for Disease Control and Prevention, Liangshan, China; ^6^ Sichuan Academy of Medical Sciences and Sichuan Provincial People’s Hospital, Chengdu, China

**Keywords:** obesity, mc4r, interaction, rural-to-urban migration, Yi people

## Abstract

**Objectives:** This study aimed to determine the association of *MC4R* gene polymorphisms (rs17782313 and rs12970134) and urbanized living environment and the gene–environment interaction with obesity in Yi people in China.

**Methods:** A 1:2 frequency-matched case–control study based on the cross-sectional data was designed. Those with BMI ≥28 kg/m^2^ were included as the case group. The age- and sex-matched controls were selected from those with BMI <24 kg/m^2^. Unconditional logistic models were used to determine the association of SNPs with obesity. Additive interaction was evaluated by calculating relative excess risk due to interaction (RERI), attributable proportion due to interaction (AP), and synergy index (SI).

**Results:** A total of 322 cases and 643 controls were included. After adjustment, allele C of rs17782313 was significantly associated with obesity (additive model, OR = 1.52, 95%CI: 1.18–1.96) in Yi people. A similar association was found in allele A of rs12970134 (additive model, OR = 1.45, 95%CI: 1.13–1.89). Yi rural-to-urban migrants were found at 2.59-fold (95%CI: 1.70–3.95) higher odds of obesity than Yi farmers. Additive interactions were found between the two SNPs and rural-to-urban migration (rs17782313: AP = 0.65, 95%CI: 0.22–1.09; rs12970134: AP = 0.59, 95%CI: 0.02–1.17).

**Conclusion:** *MC4R* gene polymorphisms positively interacted with the urbanized living environment on obesity in Yi people. The effect of the *MC4R* gene on obesity was modified by the living environment.

## Introduction

Obesity is the abnormal and excessive fat accumulation due to energy imbalance caused by increased energy intake and decreased physical activity (Burton et al., 1985). Obesity is identified as one of the vital risk factors for cardiovascular disease, diabetes mellitus, chronic kidney disease, and some types of cancer (Ni Mhurchu et al., 2004; Landsberg et al., 2013; Allott and Hursting, 2015). During the past decades, the prevalence of obesity has experienced a rapid increase in both developing and developed countries (Trends in adult body-mass, 2016).

It has been widely recognized that the obesity epidemic can be explained by the combination of physiological and behavioral factors that are triggered by the changes in the food environment and the environmentally driven reductions in physical activity (Ravussin and Ryan, 2018). Despite being exposed to the prevailing urban and rural environments, not all people become obese, suggesting the substantial genetic contribution to the regulation of body weight. It is estimated that the heritability of BMI is high, ranging from 40 to 70% (Bray et al., 2016; Heymsfield and Wadden, 2017). The melanocortin-4-receptor (MC4R), a G protein-coupled receptor, was identified to regulate adipose tissue formation and energy homeostasis (Gonçalves et al., 2018). Mutations in the *MC4R* gene were reported as the most common cause of monogenic obesity (Farooqi et al., 2003). In 2008, genome-wide association studies (GWAS) reported two common genetic variations (rs17782313 and rs12970134) near the *MC4R* gene that were associated with adiposity phenotypes in Europeans and Asians (Chambers et al., 2008; Loos et al., 2008). The association of *MC4R* variants with obesity have been replicated in Chinese (Shi et al., 2010; Huang et al., 2011; Wei et al., 2020), but the evidence in ethnic minorities was limited.

Yi people are the sixth largest ethnic minority in China residing in remote mountain areas in Southwest China, with a population of 8.71 million in 2010. In the past, Yi people lived scattered in mountainous villages, isolated from the urban areas, keeping a primitive lifestyle. Since the 1950s, some Yi farmers have been moving from rural to urban regions for living and working and subsequently transforming their lifestyle from an original rural pattern to local urban pattern (He et al., 1991). Yi farmers and rural-to-urban migrants share the same genetic background while living in diverse environments, which provides a perfect opportunity to conduct a comprehensive study on environmental and genetic factors of non-communicative diseases (He et al., 1991; Wang et al., 2010). Based on the cross-sectional survey data, our previous works have demonstrated the higher threat of obesity in rural-to-urban migrants than Yi farmers and some of the key environmental determinants in Yi people (Wang et al., 2018; Wang et al., 2020; Wang et al., 2021). At present, the role of genetic factors and their interaction with the residence environment on obesity in Yi people are needed to be illustrated. This study was designed to identify the association of *MC4R* gene polymorphisms (rs17782313 and rs12970134) with obesity in Yi people and to explore the effect of gene–environment interaction on obesity.

## Methods

### Study Population

The Yi Migrant Study was a cross-sectional survey carried out in Liangshan Yi Autonomous Prefecture, Sichuan, China, in 2015. It was designed to assess cardiovascular risk and the environmental and genetic factors in rural Yi farmers and rural-to-urban Yi migrants. A stratified cluster sampling method was used to recruit participants aged 20–80 years. Details of the sampling procedures have been described previously (Wang et al., 2018). All participants provided written informed consent before the survey.

The present study was designed as a 1:2 frequency-matched case–control study (matched for age and sex) based on the cross-sectional data. Participants with full physical examination data and blood samples were included. The minimum sample size required is calculated according to the formula for the one:c frequency matching design (Wickramaratne, 1995):

n1=(1+1c)p¯q¯(zα+zβ)2(p1−p0)2,

p1=p0OR1+p0(OR−1), p¯=(p1+cp0)(1+c),q¯=1−p¯,

$$n_{2}=n_{1}\times c.$$

In the formula, $n_{1}$ represents the sample size needed for the case group, and $n_{2}$ represents the sample size for controls; $p_{1}$ , $p_{0}$ , and OR represent the frequency of the risk allele in case and control groups, and the estimated effect value for association, respectively. According to a previous study, the allele frequency of rs17782313 in the control group was 0.2, and the OR was 2 in Chinese (Shi et al., 2010). The type I and II error rates were set at 0.05 and 0.2, respectively. Correspondingly, the minimum sample sizes for this study were 126 and 252 in case and control groups, respectively.

**FIGURE 1:**
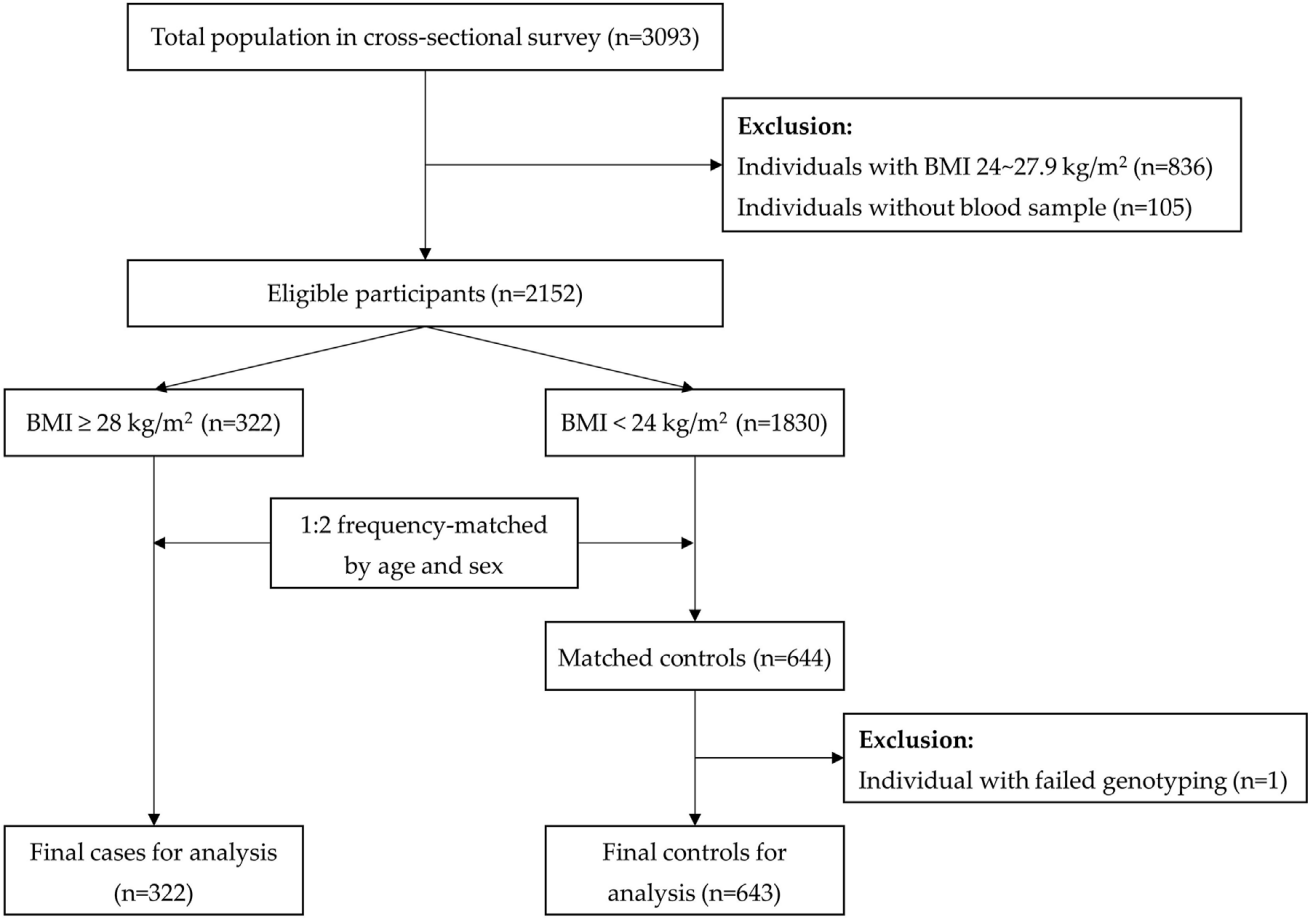
Flow chart of selection of participants in the case–control study.

In practice, those with BMI ≥28 kg/m^2^ at the survey time were all included in the case group (n = 322). The controls were selected from the people with normal weight (BMI<24 kg/m^2^) and were matched for age and sex (n = 643). In this present case–control study, overweight participants were not eligible for inclusion (Figure 1). The actual sample size was big enough to reach a conclusive result.

### Data Collection

Demographic information (age, sex, and ethnicity, etc.), socioeconomic data (education level, income, and occupation, etc.), and lifestyle factors (smoking status, drinking status, and physical activity, etc.) were collected by face-to-face interviews. The interviews were conducted by well-trained medical staff using a standard questionnaire.

The body height and weight were measured with bare feet and light clothing using a fixed stadiometer and body composition analyzer (BC-420, Tanita, Japan), with an accuracy of 0.1 cm and 0.1 kg. The average of two height measurements was recorded. The body mass index (BMI) was calculated as the weight in kilograms divided by the square of the height in meters.

### Genotyping

The genomic DNA was extracted from blood samples using the magnetic bead-based kit. A Thermo Scientific NanoDrop 2000 spectrophotometer was used to assess the purity and to determine the concentration of the extracted DNA. The *MC4R* rs17782313 (T > C) and rs12970134 (G > A) polymorphisms were genotyped using TaqMan assay. For the two SNPs, the PCR reaction was optimized in a 10 µL total volume containing 1 µL DNA template, 5 µL Master Mix, 3 µL primer, and 3 µL water.

### Definitions

Among this ethnic group, obesity was defined as BMI ≥28 kg/m^2^, according to the diagnostic criteria for the Chinese population by the Working Group on Obesity in China (Zhou, 2002).

Yi farmers were defined to be Yi people living in rural areas since birth. Yi migrants were defined to be Yi people who were born in rural areas and then migrated and had been living in an urban area for at least 1 year and were still living in the urban area.

The education level was categorized into three categories according to the last educational institution attended: illiterate, primary or middle school, and high school or above. Personal annual income was categorized into two groups in accordance to median: ≤5000 CNY and >5000 CNY. Current smoking was defined as smoking at least one cigarette per day for at least 6 months. Current drinking was defined as drinking at least twice per month with an intake of more than 640 ml (one bottle) beer or 100 ml liquor for at least 6 months. Occupational physical activity was grouped into light, moderate, and heavy according to the intensity of daily work. Leisure-time exercise was considered participation in moderate or vigorous activity for at least 20 min at leisure time, with three levels as light (<1 day per week), moderate (1–4 days per week), and heavy (5–7 days per week).

### Statistical Analysis

All analyses were conducted using SAS statistical software (version 9.4; SAS Institute Inc., Cary, NC, USA). A two-tailed *p*-value of <0.05 was considered statistically significant for all analyses.

Categorical variables were presented as frequency and percentage, and the differences between groups were compared using chi-square tests. The Hardy–Weinberg equilibrium (HWE) was tested to assess the deviation between observed and expected frequencies using chi-square tests in the control group. Also, chi-square tests were used to compare genotypes and allele frequencies between the obesity cases and the controls.

To determine the association between *MC4R* gene polymorphisms with obesity, unconditional logistic regression models were applied to test all four inheritance models for each variant (dominant, recessive, codominant, and additive). All models were adjusted by age, sex, socioeconomic status, and lifestyle factors.

The term of interaction refers to the biological interaction of two or more causes of disease that together assert their influence on disease risk and can be assessed by multiplicative and additive models in epidemiology studies. The general consensus is that measuring interaction on the additive scale is the most appropriate for assessing the public health importance of interactions (Rothman, 1974; Rothman et al., 1980; Ahlbom and Alfredsson, 2005). In this study, both multiplicative and additive interactions were evaluated to examine the interaction between *MC4R* SNPs and dwelling environment on obesity. In multiplicative models, product terms between SNPs and rural-to-urban migration were added in the regressions. The additive interactions were evaluated by the relative excess risk due to interaction (RERI), attributable proportion due to interaction (AP), and synergy index (SI), which have been systematically introduced by Rothman (Rothman, 1974; Rothman, 1976) and were defined as the relationship between factors, which exhibit a joint effect that exceeds the sum of the separate effects. RERI, AP, and SI were calculated using the regression coefficients and covariance matrix obtained from the multivariable logistic regression models (Andersson et al., 2005). Odds ratios (ORs) of SNPs and migration were calculated through the logistic regression models as the approximate value of relative risk (RR), since these measures of RERI, AP, and SI are commonly based on RRs. Then, RERI, AP, and SI were calculated with the formulas: RERI = OR_11_ - OR_10_ - OR_01_ + 1, AP = RERI/OR_11_, and SI= (OR_11_–1)/[(OR_01_–1) + (OR_10_–1)]. The delta method introduced by Hosmer and Lemeshow was used to calculate the 95% confidence interval (CI) of RERI, AP, and SI (Hosmer and Lemeshow, 1992; Andersson et al., 2005). The bootstrap method (Assmann et al., 1996) was also used for comparison. If the 95%CIs of RERI and AP did not include 0 and the 95%CI of SI did not include 1, the interaction was present.

## Results

A total of 3,093 Yi people were investigated in the cross-sectional survey, among whom 322 were obese cases. With a 1:2 frequency matching process, 644 controls with BMI<24 kg/m^2^ were eligible for inclusion. One of the controls with failed genotyping was excluded. Finally, 965 Yi people were included for the final analysis. The flow of selecting the study subjects is shown in Figure 1.

The characteristics of the study subjects are summarized in Table 1. Among 965 Yi people, 588 were Yi farmers, and 377 were rural-to-urban migrants. Compared with the control group, cases had a high education and income level. Except for the drinking status, the lifestyle factors between the case and control groups were significantly different. Stratified descriptions of the characteristics of Yi farmers and migrants are also shown in Table 1. A comparison of the demographic characteristics was also conducted between the selected controls and non-selected eligible participants (see Supplementary Table S1). The characteristics between the two groups were comparable except for age group and education.

**TABLE 1:** Basic characteristics of the case and control groups in Yi people.

		Total			Yi farmers			Yi migrants		
		Case	Control	*P*	Case	Control	*P*	Case	Control	*P*
		N = 322	N = 643		N = 126	N = 462		N = 196	N = 181	
Sex, n (%)				0.9872			0.3644			0.1738
	Men	107 (33.23)	214 (33.28)		39 (30.95)	163 (35.28)		68 (34.69)	51 (28.18)	
	Women	215 (66.77)	429 (66.72)		87 (69.05)	299 (64.72)		128 (65.31)	130 (71.82)	
Age (years), n (%)				0.9999			0.0761			0.9911
	20–29	20 (6.21)	40 (6.22)		12 (9.52)	31 (6.71)		8 (4.08)	9 (4.97)	
	30–39	69 (21.43)	137 (21.31)		32 (25.40)	101 (21.86)		37 (18.88)	36 (19.89)	
	40–49	109 (33.85)	218 (33.90)		51 (40.48)	166 (35.93)		58 (29.59)	52 (28.73)	
	50–59	75 (23.29)	150 (23.33)		25 (19.84)	104 (22.51)		50 (25.51)	46 (25.41)	
	60–80	49 (15.22)	98 (15.24)		6 (4.76)	60 (12.99)		43 (21.94)	38 (20.99)	
Education, n (%)				<0.0001			0.2532			0.2052
	Illiterate	155 (48.14)	409 (63.61)		85 (67.46)	333 (72.08)		70 (35.71)	76 (41.99)	
	Primary or middle school	106 (32.92)	188 (29.24)		36 (28.57)	121 (26.19)		70 (35.71)	67 (37.02)	
	High school or above	61 (18.94)	46 (7.15)		5 (3.97)	8 (1.73)		56 (28.57)	38 (20.99)	
Income (CNY/y), n (%)				<0.0001			0.0337			0.7168
	<5,000	121 (37.58)	395 (61.43)		87 (69.05)	361 (78.14)		34 (17.35)	34 (18.78)	
	≥5,000	201 (62.42)	248 (38.57)		39 (30.95)	101 (21.86)		162 (82.65)	147 (81.22)	
Smoking status, n (%)				0.0006			0.0840			0.1358
	Never	221 (68.63)	424 (65.94)		85 (67.46)	299 (64.72)		136 (69.39)	125 (69.06)	
	Former	21 (6.52)	14 (2.18)		6 (4.76)	8 (1.73)		15 (7.65)	6 (3.31)	
	Current	80 (24.85)	205 (31.88)		35 (27.78)	155 (33.55)		45 (22.96)	50 (27.62)	
Drinking status, n (%)				0.2919			0.5411			0.0531
	Never	201 (62.42)	434 (67.50)		84 (66.67)	305 (66.02)		117 (59.69)	129 (71.27)	
	Former	29 (9.01)	51 (7.93)		14 (11.11)	39 (8.44)		15 (7.65)	12 (6.63)	
	Current	92 (28.57)	158 (24.57)		28 (22.22)	118 (25.54)		64 (32.65)	40 (22.10)	
Occupational physical activity, n (%)				<0.0001			0.3837			0.4080
	Light	188 (58.39)	264 (41.06)		31 (24.60)	129 (27.92)		157 (80.10)	135 (74.59)	
	Moderate	38 (11.80)	56 (8.71)		11 (8.73)	26 (5.63)		27 (13.78)	30 (16.57)	
	Heavy	96 (29.81)	323 (50.23)		84 (66.67)	307 (66.45)		12 (6.12)	16 (8.84)	
Leisure-time exercise, n (%)				<0.0001			0.0080			0.1079
	Light	179 (55.59)	516 (80.25)		110 (87.30)	434 (93.94)		69 (35.20)	82 (45.30)	
	Moderate	61 (18.94)	54 (8.40)		9 (7.14)	9 (1.95)		52 (26.53)	45 (24.86)	
	Heavy	82 (25.47)	73 (11.35)		7 (5.56)	19 (4.11)		75 (38.27)	54 (29.83)	

As Table 2 shows, in Yi people, the genotype and allele frequencies of rs17782313 and rs12970134 in the case and control groups were significantly different (*p* < 0.05). Stratification analysis shows that in Yi farmers, only the rs17782313 allele frequencies (T/C) differed in the case and control groups. Comparison in Yi migrants shows the same results as among all Yi people. In addition, the two SNPs exhibited HWE in all tests (*p* > 0.05).

**TABLE 2:** Comparison of genotype and allele frequencies and the HWE test between case and control groups in Yi people.

	Total				Yi farmer				Yi migrant			
	Case	Control	*P*	HWE	Case	Control	*P*	HWE	Case	Control	*P*	HWE
	N = 322	N = 643		*P*	N = 126	N = 462		*P*	N = 196	N = 181		*P*
rs17782313												
Genotype			0.0004	0.8919			0.1204	0.8674			0.0065	0.9871
T/T	193 (59.94)	448 (69.67)			76 (60.32)	317 (68.61)			117 (59.69)	131 (72.38)		
T/C	106 (32.92)	178 (27.68)			43 (34.13)	132 (28.57)			63 (32.14)	46 (25.41)		
C/C	23 (7.14)	17 (2.64)			7 (5.56)	13 (2.81)			16 (8.16)	4 (2.21)		
Allele			0.0002				0.0445				0.0013	
T	492 (76.40)	1,074 (83.51)			195 (77.38)	766 (82.90)			297 (75.77)	308 (85.08)		
C	152 (23.60)	212 (16.49)			57 (22.62)	158 (17.10)			95 (24.23)	54 (14.92)		
rs12970134												
Genotype			0.0018	0.9611			0.0676	0.8723			0.0196	0.8468
G/G	206 (63.98)	458 (71.23)			82 (65.08)	325 (70.35)			124 (63.27)	133 (73.48)		
G/A	94 (29.19)	169 (26.28)			35 (27.78)	124 (26.84)			59 (30.10)	45 (24.86)		
A/A	22 (6.83)	16 (2.49)			9 (7.14)	13 (2.81)			13 (6.63)	3 (1.66)		
Allele			0.0016				0.0740				0.0067	
G	506 (78.57)	1,085 (84.37)			199 (78.97)	774 (83.77)			307 (78.32)	311 (85.91)		
A	138 (21.43)	201 (15.63)			53 (21.03)	150 (16.23)			85 (21.68)	51 (14.09)		

**FIGURE 2:**
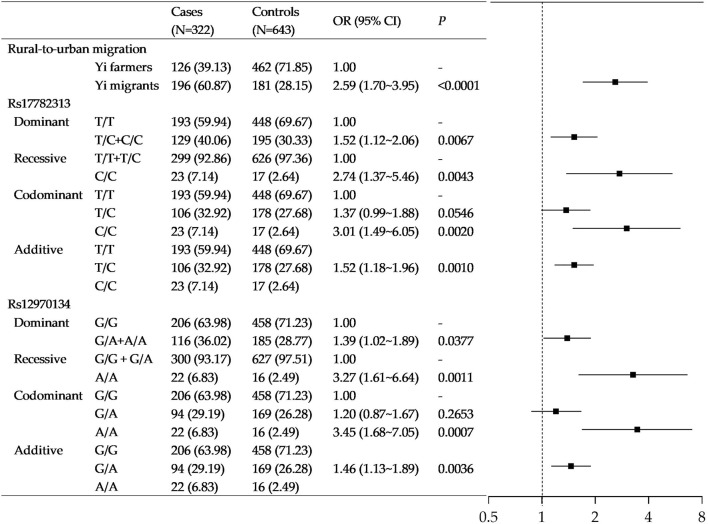
Association of rural-to-urban migration and *MC4R* gene polymorphisms with obesity in Yi people. Note that the models were adjusted for age, sex, education, income, smoking status, drinking status, occupational physical activity, leisure-time exercise, and rural-to-urban migration.

Figure 2 shows the association of rural-to-urban migration and *MC4R* gene polymorphism with obesity in Yi people. With adjustment for age, sex, socioeconomic status, and lifestyle factors, compared with Yi farmers, the odds ratio for rural-to-urban Yi migrants of obesity was 2.59 (95%CI: 1.70–3.95).

The polymorphism of rs17782313 was significantly associated with obesity in Yi people under all four inheritance modes. In particular, those carrying homozygote C/C had 3.01-fold (95%CI: 1.49–6.05) higher odds of obesity than those with genotype T/T. As additive mode shows, the odds of obesity increased by 0.52 folds for each additional risk allele (OR = 1.52, 95%CI: 1.18–1.96). Likewise, a significant association was also found between rs12970134A > G variation with obesity (see Figure 2).

To test the interaction between *MC4R* gene polymorphism and rural-to-urban migration, both multiplicative and additive interaction models based on dominant and recessive inheritance modes were evaluated. The results showed that only the additive interaction based on the recessive inheritance model was significant (as shown in Table 3). For rs17782313 polymorphism, Yi migrants with genotype C/C were at 10-fold higher odds of obesity relative to Yi farmers with genotypes T/T and T/C, with AP and its 95%CI being 0.65 (0.22–1.09). However, the RERI was not statistically significant, although it is closely related to AP. Similar results were found in the detection of rs12970134 polymorphism. The results of the additive interaction models based on dominant inheritance models and multiplicative interaction models are shown in Supplementary Tables 2–4. The results of the bootstrap method are shown in Supplementary Table S5 and indicate similar results as shown here.

**TABLE 3:** Additive interaction between *MC4R* gene polymorphisms and rural-to-urban migration based on the recessive inheritance mode.

	Gene	Migration	Case	Control	OR (95%CI)	*P*
			N = 322	N = 643		
rs17782313						
OR_00_	0	0	119 (36.96)	449 (69.83)	1.00	-
OR_10_	1	0	7 (2.17)	13 (2.02)	2.02 (0.78–5.28)	0.1491
OR_01_	0	1	180 (55.90)	177 (27.53)	2.46 (1.60–3.80)	<0.0001
OR_11_	1	1	16 (4.97)	4 (0.62)	10.03 (3.12–32.23)	0.0001
				RERI	6.54 (-5.03–18.12)	
				AP	**0.65 (0.22∼1.09)**	
				SI	3.63 (0.82–15.93)	
rs12970134						
OR_00_	0	0	117 (36.34)	449 (69.83)	1.00	-
OR_10_	1	0	9 (2.80)	13 (2.02)	2.86 (1.17–6.96)	0.0209
OR_01_	0	1	183 (56.83)	178 (27.68)	2.54 (1.65–3.90)	<0.0001
OR_11_	1	1	13 (4.04)	3 (0.47)	10.80 (2.87–40.64)	0.0004
				RERI	6.40 (-7.88–20.69)	
				AP	**0.59 (0.02∼1.17)**	
				SI	2.88 (0.58–14.40)	

Note: for rs17782313, gene-0 stands for genotypes T/T and T/C, and gene-1 stands for genotype C/C. For rs12970134, gene-0 stands for genotypes G/G and G/A, and gene-1 stands for genotype A/A. Migration-0 stands for Yi farmers, and Migration-1 stands for Yi migrants. RERI: relative excess risk due to interaction; AP: attributable proportion due to interaction; SI: synergy index. Models were adjusted for age, sex, education, income, smoking status, drinking status, occupational physical activity, and leisure-time exercise.

Bold values: significant interaction.

**FIGURE 3:**
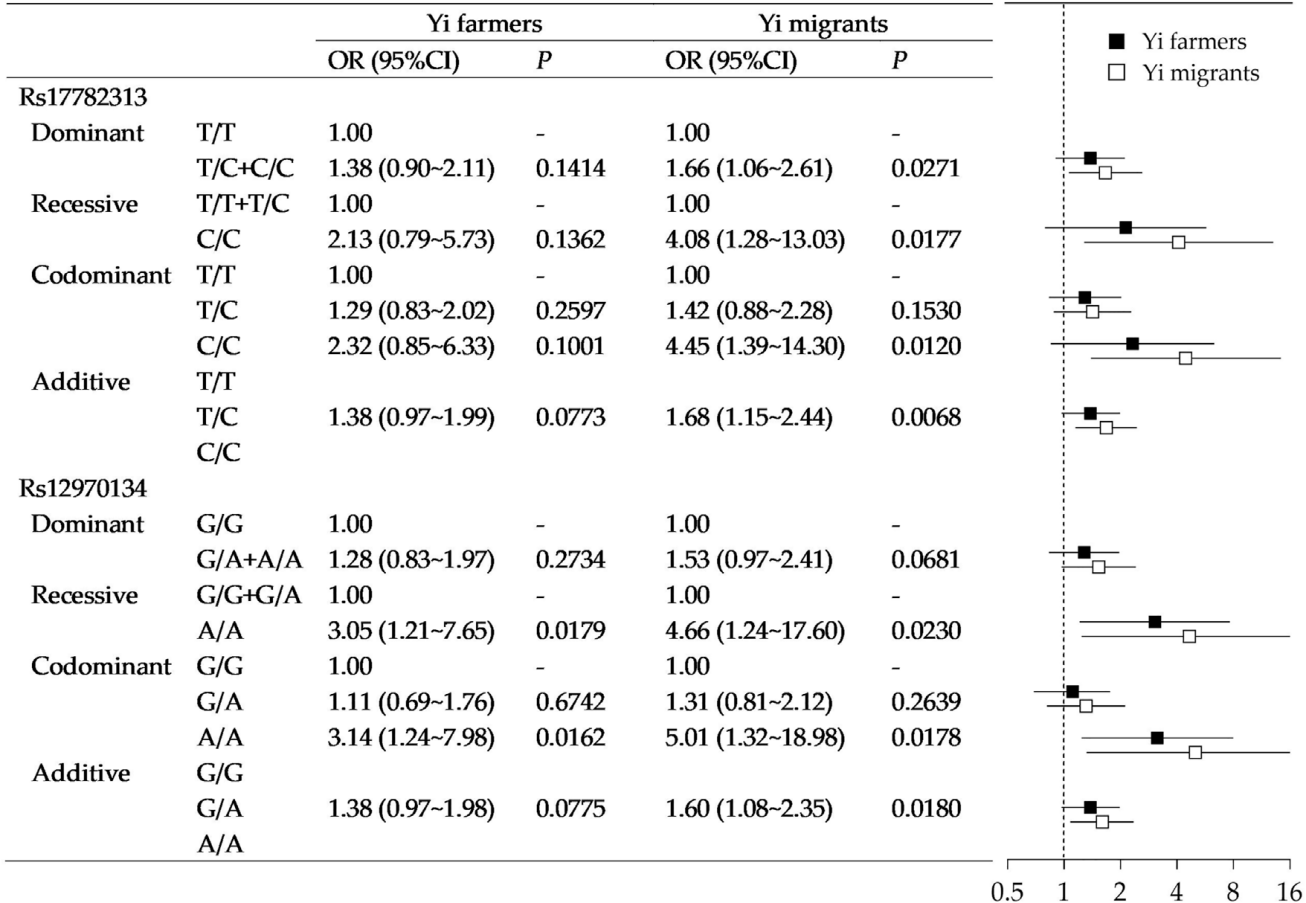
Association of *MC4R* gene polymorphisms with obesity in Yi farmers and Yi migrants. Note that the models were adjusted for age, sex, education, income, smoking status, drinking status, occupational physical activity, and leisure-time exercise.

Stratified analysis was then performed in Yi farmers and Yi rural-to-urban migrants. Figure 3 shows a heterogeneous association between *MC4R* gene polymorphism with obesity in the two groups. For rs17782313, with adjustment for the covariant, no significant association was observed in Yi farmers. While in Yi migrants, significances existed, and all the point estimates tended to be more apparent than Yi farmers. Similarly, rs17782313 polymorphism in Yi migrants had a stronger association with obesity than Yi farmers.

## Discussion

With a population-based case–control design, this study confirmed the association between *MC4R* gene polymorphism and obesity as well as the interaction with rural-to-urban living environment transformation in Yi people. After adjusting for age, sex, socioeconomic factors, and lifestyle factors, the C allele of rs17782313 and A allele of rs12970134 were related to obesity in Yi people. This study also suggested a significantly higher risk of obesity in Yi migrants than Yi farmers, which has been proved in our previous cross-sectional analysis (Wang et al., 2021).

The association between the SNPs near the *MC4R* gene and obesity was first reported in European populations and has subsequently been verified in Finland and France (Cauchi et al., 2009), Denmark (Thomsen et al., 2012), Japan (Hotta et al., 2009), and other different areas. Grant et al. (Grant et al., 2009) found that the *MC4R* gene polymorphism was associated with obesity in European–American children but not in African–American children. In 2010, Huang et al.(Huang et al., 2011) verified the association between *MC4R* gene polymorphism and obesity in Chinese. The results showed that each additional C allele in rs17782313 increased the risk of obesity by 39%, and genotype CC and CT increased the risk by 87% and 44% compared with the TT genotype, respectively. In contrast, another cohort study in Hong Kong did not find a significant association between rs17782313 and central obesity (waist circumference >90 cm in men and >80 cm in women) (Cheung et al., 2011). Studies among ethnic minorities in China are limited. A study conducted on Maonan people in Guangxi Province examined the association of the *MC4R* gene with obesity, and the results showed that rs17782313 and rs476828 of the three SNPs were related to obesity (Wei et al., 2020).

Based on the four inheritance modes, the homozygous risk allele’s genotypes of the two SNPs (CC and AA) had the highest odds of obesity, while the heterozygous genotypes were not significantly associated with obesity in the codominant modes. A meta-analysis also revealed that only the homozygous genotype of rs17782313 was significantly associated with obesity in the codominant model (Yu et al., 2020), which was consistent with our study. The results suggested that the *MC4R* gene may inherit in the recessive mode.

The complex network of environmental and genetic factors contributes to the occurrence of obesity. In parallel with successful gene identifications, the number of gene–environment interaction studies has grown rapidly (Reddon et al., 2016). Interaction indicates the mutual synergy or antagonism of two factors, which is also known as effect modification. The statistical interaction between two or more risk factors is evaluated by the coefficient of the product term. Interaction is thus measured in terms of departure from a multiplicative (Hunter, 2005; Franks et al., 2013). Alternatively, the additive interaction model (also called biological interaction) between two factors is defined as their co-participation in the same causal mechanism to disease development (Andersson et al., 2005; Ding et al., 2011; Reddon et al., 2016). The UK Biobank estimated that the 376 SNPs could explain 5.2% of the BMI variation, and gene–environment interaction can explain 1.9% of it (Sulc et al., 2020). By constructing the genetic risk score with 69 SNPs, the study found the interaction between genes and environmental factors, including socioeconomic indicators, the length of TV watching, and physical activity. Compared with people in a low obesity-prone environment, people in a high obesity-prone environment had high weight by 0.9 kg for every 10 more obesity-related alleles (Tyrrell et al., 2017). The gene–environment interaction studies not only help explain the underlying mechanism of disease occurrence but also provide a basis for eliminating environmental risk factors.

In the current study, an additive interaction was detected between the *MC4R* gene polymorphism and urbanized living environment (evaluated by rural-to-urban migration). We detected statistically significant AP values in the two recessive inheritance models. Among rural-to-urban migrants carrying risk genotypes, it was estimated that approximately 60% of the obesity risk could be attributed to the gene–environment interaction. Notably, neither RERI nor SI was significant, although RERI is closely related to AP. The inconsistency indicated that the results should be prudently interpreted. The limited sample size may restrict the statistical power, as it can be seen that along with the statistical insignificance of RERI and SI, the CIs for AP were quite wide (0.22–1.09, 0.02–1.17). A few methods have been developed to calculate the confidence intervals for interaction measures, and there is no definite agreement on which computational methods are the most appropriate (Ding et al., 2011). There is a concern that the delta method may have the consequence of false-positive results (Zou, 2008). In addition to the delta method, we also used the bootstrap re-sampling method that was introduced by Assmann (Assmann et al., 1996). The results are shown in Supplementary Table S5, and APs are consistently significant.

A subsequent stratification analysis illustrated the differed association between *MC4R* gene and obesity in Yi farmers and Yi rural-to-urban migrants. In this genetically homogenous population, the *MC4R* gene played a stronger role in rural-to-urban migrants than originally residing farmers. Our results could reflect lifestyle differences between Yi farmers and migrants and also suggested that the obesity-prone urban environment may provide favorable conditions for the expression of gene function. The *MC4R* gene was identified to play a role in body weight through regulating the appetite and metabolic homeostasis. In previous studies, the *MC4R* gene has been found to interact with diet (Crovesy and Rosado, 2019; Mousavizadeh et al., 2020). The increased availability of high-calorie foods in urban areas compared to rural areas may explain the interaction between the *MC4R* gene and rural-to-urban migration.

The selection of the controls in a case–control study is a fundamental step. This study was a re-sampling set based on cross-sectional data, and we compared the basic characteristics of the selected controls and non-selected eligible participants (Supplementary Table S1). The disparity in the age group is reasonable due to the fact that there was a difference in age between normal-weight and obesity groups in the cross-sectional data. In order to match the age in the case–control study, unequal probability sampling was conducted among the eligible population. The unbalanced education level could also be explained by the disparity in age, since the younger participants were more educated.

One of the strengths of this study lies in the collection of data from two groups of people with the same genetic background while in diverse living environments. The sample allows us to conduct a comprehensive study to assess the genetic and environmental factors of obesity and other chronic diseases. In addition, the inclusion criteria of the control group minimized the bias of misclassification. The major limitation of the study is the lack of data on diet. Due to the difficulty in obtaining reliable and accurate dietary information in a large-scale population-based field survey, data on dietary were not available in this study. We also acknowledge that obesity is polygenic in nature, while we only focused on *MC4R* in this study, which has a minor contribution to obesity. Finally, we found the interaction between the gene and urbanized environment, while rural-to-urban migration is an indicating factor, reflecting a comprehensive lifestyle aspect of the urban environment. Further investigations are needed to enable insight into the relative contributions of each aspect.

## Conclusion

Our findings suggested that both the gene and urbanized living environment played a significant role in obesity in Yi people. *MC4R* gene polymorphisms positively interacted with rural-to-urban migration on obesity in Yi people. These findings emphasize the importance of healthy lifestyle adherence for obesity prevention in the era of urbanization in China.

## Data Availability

The raw data supporting the conclusion of this article will be made available by the authors, without undue reservation.
