# Supplementary material for: MC4R Gene Polymorphisms Interact With the Urbanized Living Environment on Obesity: Results From the Yi Migrant Study
Source: Front Genet. 2022 Apr 14;13:849138. doi: 10.3389/fgene.2022.849138 (PMC9046839; doi:10.3389/fgene.2022.849138)
Supplement: Supplementary file 1 [file Table1.DOCX]

**Supplementary table 1. Comparison of demographic characteristics between selected controls and non-selected eligible participants**

|  |  | Selected  N=643 | Non-selected  N=1274 | *P* |
| --- | --- | --- | --- | --- |
| Sex, n (%) | |  |  | 0.7576 |
|  | Men | 214 (33.28) | 433 (33.99) |  |
|  | Women | 429 (66.72) | 841 (66.01) |  |
| Age (years), n (%) | |  |  | <0.0001 |
|  | 20~29 | 40 (6.22) | 178 (13.97) |  |
|  | 30~39 | 137 (21.31) | 342 (26.84) |  |
|  | 40~49 | 218 (33.90) | 281 (22.06) |  |
|  | 50~59 | 150 (23.33) | 149 (11.70) |  |
|  | 60~80 | 98 (15.24) | 324 (25.43) |  |
| Education, n (%) | |  |  | 0.0398 |
|  | Illiterate | 409 (63.61) | 747 (58.63) |  |
|  | Primary or middle school | 188 (29.24) | 398 (31.24) |  |
|  | High school or above | 46 (7.15) | 129 (10.13) |  |
| Income (CNY/y), n (%) | |  |  | 0.1905 |
|  | <5000 | 395 (61.43) | 743 (58.32) |  |
|  | ≥5000 | 248 (38.57) | 531 (41.68) |  |
| Smoking status, n (%) | |  |  | 0.2983 |
|  | Never | 424 (65.94) | 845 (66.32) |  |
|  | Former | 14 (2.18) | 43 (3.38) |  |
|  | Current | 205 (31.88) | 386 (30.30) |  |
| Drinking status, n (%) | |  |  | 0.5047 |
|  | Never | 434 (67.50) | 847 (66.48) |  |
|  | Former | 51 (7.93) | 88 (6.91) |  |
|  | Current | 158 (24.57) | 339 (26.61) |  |
| Occupational physical activity, n (%) | |  |  | 0.9533 |
|  | Light | 264 (41.06) | 514 (40.35) |  |
|  | Moderate | 56 (8.71) | 111 (8.71) |  |
|  | Heavy | 323 (50.23) | 649 (50.94) |  |
| Leisure-time exercise, n (%) | |  |  | 0.6744 |
|  | Light | 516 (80.25) | 1034 (81.23) |  |
|  | Moderate | 54 (8.40) | 111 (8.72) |  |
|  | Heavy | 73 (11.35) | 128 (10.05) |  |

**Supplementary table 2. Additive interaction between *MC4R* gene polymorphism and rural-to-urban migration based on dominant inheritance mode**

|  | Gene | Migration | Cases  N=322 | Controls  N=643 | OR (95%CI) | *P* |
| --- | --- | --- | --- | --- | --- | --- |
| Rs17782313 |  |  |  |  |  |  |
| OR_00_ | 0 | 0 | 76 (23.60) | 317 (49.30) | 1.00 | - |
| OR_10_ | 1 | 0 | 50 (15.53) | 145 (22.55) | 1.41 (0.93~2.13) | 0.1075 |
| OR_01_ | 0 | 1 | 117 (36.34) | 131 (20.37) | 2.46 (1.54~3.95) | 0.0002 |
| OR_11_ | 1 | 1 | 79 (24.53) | 50 (7.78) | 4.10 (2.38~7.08) | <0.0001 |
|  |  |  |  | RERI | 1.23 (-0.55~3.02) |  |
|  |  |  |  | AP | 0.30 (-0.03~0.63) |  |
|  |  |  |  | SI | 1.66 (0.85~3.24) |  |
| Rs12970134 |  |  |  |  |  |  |
| OR_00_ | 0 | 0 | 82 (25.47) | 325 (50.54) | 1.00 | - |
| OR_10_ | 1 | 0 | 44 (13.66) | 137 (21.31) | 1.27 (0.83~1.94) | 0.2676 |
| OR_01_ | 0 | 1 | 124 (38.51) | 133 (20.68) | 2.44 (1.54~3.89) | 0.0002 |
| OR_11_ | 1 | 1 | 72 (22.36) | 48 (7.47) | 3.74 (2.16~6.49) | <0.0001 |
|  |  |  |  | RERI | 1.03 (-0.63~2.69) |  |
|  |  |  |  | AP | 0.27 (-0.07~0.62) |  |
|  |  |  |  | SI | 1.60 (0.79~3.24) |  |
| Note: For rs17782313, gene-0 stands for genotypes T/T, gene-1 stands for genotype T/C and C/C. For rs12970134, gene-0 stands for genotypes G/G, gene-1 stands for genotype G/A and A/A. Migration-0 stands for Yi farmers, migration-0 stands for Yi migrants. RERI: relative excess risk due to interaction. AP: attributable proportion due to interaction. SI: synergy index. Models were adjusted for age, sex, education, income, smoking status, drinking status, occupational physical activity, and leisure-time exercise. | | | | | | |

**Supplementary table 3. Multiplicative interaction between *MC4R* gene polymorphism and rural-to-urban migration based on dominant inheritance mode**

|  | Cases  N=322 | Controls  N=643 | β | OR (95%CI) | *P* |
| --- | --- | --- | --- | --- | --- |
| Rs17782313 |  |  |  |  |  |
| T/C+C/C | 129 (40.06) | 195 (30.33) | 0.34 | 1.41 (0.93~2.13) | 0.1075 |
| Migration | 196 (60.87) | 181 (28.15) | 0.90 | 2.46 (1.54~3.95) | 0.0002 |
| (T/C+C/C) × Migration |  |  | 0.17 | 1.19 (0.64~2.18) | 0.5856 |
| Rs12970134 |  |  |  |  |  |
| G/A+A/A | 116 (36.02) | 185 (28.77) | 0.24 | 1.27 (0.83~1.94) | 0.2676 |
| Migration | 196 (60.87) | 181 (28.15) | 0.89 | 2.44 (1.53~3.89) | 0.0002 |
| (G/A+A/A) × Migration |  |  | 0.19 | 1.21 (0.65~2.24) | 0.5548 |
| Note: Models were adjusted for sex, age, education, income, smoking status, drinking status, occupational physical activity, and leisure-time exercise. | | | | | |

**Supplementary table 4. Multiplicative interaction between *MC4R* gene polymorphism and rural-to-urban migration based on recessive inheritance mode**

|  | Cases  N=322 | Controls  N=643 | β | OR (95%CI) | *P* |
| --- | --- | --- | --- | --- | --- |
| Rs17782313 |  |  |  |  |  |
| C/C | 23 (7.14) | 17 (2.64) | 0.71 | 2.02 (0.78~5.27) | 0.1491 |
| Migration | 196 (60.87) | 181 (28.15) | 0.90 | 2.46 (1.60~3.80) | <0.0001 |
| C/C × Migration |  |  | 0.70 | 2.01 (0.45~8.93) | 0.3580 |
| Rs12970134 |  |  |  |  |  |
| A/A | 22 (6.83) | 16 (2.49) | 1.05 | 2.86 (1.17~6.96) | 0.0209 |
| Migration | 196 (60.87) | 181 (28.15) | 0.93 | 2.54 (1.65~3.90) | <0.0001 |
| A/A × Migration |  |  | 0.40 | 1.49 (0.31~7.23) | 0.6209 |
| Note: Models were adjusted for sex, age, education, income, smoking status, drinking status, occupational physical activity, and leisure-time exercise. | | | | | |

**Supplementary table 5. Point estimates and confidence intervals for additive interaction between *MC4R* gene polymorphism and rural-to-urban migration based on recessive inheritance mode by the bootstrap methods**

|  | Point estimates | 95% Confidence intervals | | |
| --- | --- | --- | --- | --- |
|  |  | BSE | BP1 | BP2 |
| Rs17782313 |  |  |  |  |
| RERI | 6.54 | -5.61~18.69 | 1.65~27.79 | -14.71~11.43 |
| AP | 0.65 | 0.37~0.93 | 0.30~0.89 | 0.41~1.00 |
| SI | 3.63 | -1.83~9.09 | 1.58~12.85 | -5.59~5.68 |
| Rs12970134 |  |  |  |  |
| RERI | 6.40 | -6.44~19.24 | 1.14~25.65 | -12.85~11.66 |
| AP | 0.59 | 0.23~0.95 | 0.18~0.84 | 0.34~1.00 |
| SI | 2.88 | -1.71~7.47 | 1.28~8.25 | -2.49~4.48 |
| Two hundred bootstrap re-sampling was performed with n=700 in each sample.  RERI: relative excess risk due to interaction; AP: attributable proportion due to interaction; SI: synergy index; BSE: bootstrap standard error technique; BP1: bootstrap method 1; BP2: bootstrap method 2. Confidence intervals in BSE, BP1, and BP2 were calculated by the following formula:  BSE: $\hat{X}\pm1.96\cdot\sqrt{\hat{V}ar(X^{*})}$ ,  where $\hat{X}$ is the point estimate for interaction in the original analysis, and $\hat{V}ar(X^{*})$ is the sample variance of the point estimate using the bootstrap sampling method.  BP1: ($X_{0.025}^{*},X_{0.975}^{*}$) ,  where $X_{0.025}^{*} \mathrm{and} X_{0.975}^{*}$ denote the 2.5^th^ and 97.5^th^ percentiles of the estimates distribution using the bootstrap sampling method.  BP2: (${2\hat{X}-X}_{0.975}^{*},{2\hat{X}-X}_{0.025}^{*}$) | | | | |
